# Changes in free-roaming dog population demographics and health associated with a catch-neuter-vaccinate-release program in Jamshedpur, India

**DOI:** 10.1371/journal.pone.0317636

**Published:** 2025-10-07

**Authors:** Lauren Margaret Smith, Tamara Kartal, Sanjay Rawat, Amit Chaudhari, Ashok Kumar, Rajesh Kumar Pandey, Rupert Julian Quinnell, Lisa M. Collins

**Affiliations:** 1 Faculty of Biological Sciences, University of Leeds, Leeds, United Kingdom; 2 The Walter and Eliza Hall Institute of Medical Research, Melbourne, Victoria, Australia; 3 Department of Medical Biology, University of Melbourne, Melbourne, Victoria, Australia; 4 Humane World for Animals, Washington, District of Columbia, United States of America; 5 University of Surrey, Guildford, Surrey, United Kingdom; West Bengal University of Animal and Fishery Sciences, INDIA

## Abstract

India’s large free-roaming dog populations contribute to significant human health, environmental, and social challenges. Population management strategies, such as catch-neuter-vaccinate-release (CNVR), aim to reduce dog numbers, improve their welfare, and reduce human-animal conflict. Humane Society International (HSI; now operating as Humane World for Animals), in partnership with the Animal Health Foundation, implemented a CNVR program in Jamshedpur, neutering and vaccinating over 20,000 dogs. This study evaluated the impact of this program on dog health, population structure and size. The study areas encompassed 10 sites within Jamshedpur, including both intervention sites where CNVR was directly applied and sites without direct intervention. Data was collected from May 2014 until December 2018, including bi-annual street surveys, as well as clinical data from the dogs captured and treated. We fit logistic regression, negative binomial, and binomial mixed effects models to assess changes in dog population characteristics, health, and reproductive conditions over time in relation to the CNVR intervention. We found that, over the period of this study, the probability of dogs entering the clinic with mange, transmissible venereal tumours, and pregnant significantly reduced. Street surveys showed an increase in sterilised dogs, with higher proportions observed in CNVR-treated sites, although the counts of dogs observed increased overall. The age-structure of free-roaming dogs remained stable over time. In CNVR-treated areas, the probability of observing lactating female dogs decreased, whereas it increased in untreated sites. This work contributes to the growing body of knowledge investigating the impact of dog population management interventions. Continued monitoring and evaluation of CNVR programs are required to identify optimal coverage required to reduce population size effectively.

## 1. Introduction

The worldwide population of domestic dogs (*Canis familiaris*) is estimated to be between 700 million to 1 billion [1,2], a high proportion of which are “free-roaming” for all, or at least part of, the day [1]. Free-roaming dogs can roam and reproduce without restriction, with possible consequences for public health [3–5], wildlife conservation [6–8], and livestock losses [9–13]. Free-roaming dogs may also experience high mortality and poor welfare [14–17]. India has high densities of free-roaming dogs, with estimates in rural areas as high as 719 dogs per km^2^ [18]. Consequently, the country faces serious public health, environmental, and social challenges. There are an estimated 20 million dog bites in India per year (approximately 150 bites per 10,000 people) [19], and victims often require medical attention or post-exposure rabies prophylaxis. Dogs are responsible for 99% of human-rabies transmissions and, although the true rabies burden in India is unknown, it is estimated to result in 18,000–20,000 human deaths per year (approximately 35% of the reported worldwide rabies burden) [19,20]. Free-roaming dogs are also responsible for the killing of livestock, such as sheep, goats, and donkeys, contributing to substantial economic losses, and exacerbating human-wildlife conflict, as predation by dogs can be mistaken for predation by wolves or snow leopards [9,21]. There are also beneficial aspects of the free-roaming dog population: dogs may provide companionship, guard livestock, and reduce vermin.

In India, the free-roaming dog population is dependent on humans as a source of food, either directly by households providing food, or indirectly, for example, through improper refuse disposal [22]. It is estimated that free-roaming dogs in India have a short lifespan of less than four years [23], and that only 18% of puppies survive to one year old [24,25]. Birth rates are high and almost 50% of female dogs are estimated to become pregnant in any given year [23]. These high mortality and birth rates lead to high population turnover, which makes maintaining high vaccination coverages challenging for infectious disease control, such as rabies [26].

Dog population management can reduce the population size or the risks associated with free-roaming dogs, and improve the health and welfare of the dog population, depending on the methods employed. Dog population management methods can include culling, sheltering, fertility control, reducing the carrying capacity of the population (e.g., by reducing the availability of resources [22]), and encouraging responsible ownership practices [27]. In India, national law requires that street or community owned (i.e., free-roaming) dogs are managed through the Animal Birth Control Rules of 2023 [28]. This law stipulates that free-roaming dogs must be managed through fertility control, where dogs are caught, neutered, vaccinated against rabies (i.e., catch-neuter-vaccinate-release; CNVR), and returned to the same area where they were captured, once they complete the mandatory post-operative care period of three days.

Several studies have attempted to evaluate the impact of dog population management strategies, reviewed by [27]. In India, the reported impact of fertility control on the health and welfare of free-roaming dogs varies, with both positive [29] and negative [29,30] impacts. This includes reports of improved body condition scores [31,32] and reduced presence of visible injuries [32], and both increased and decreased prevalence of pathogens and visible skin conditions [32]. These effects have been attributed to a lack of sex hormones, which may reduce sexual competition and mate-seeking behaviour, which in turn can lead to decreased aggression [33] and improved body conditions [34,35]. Fertility control programs, such as CNVR, regularly involve vaccination and antiparasitic treatments, which may also improve health and welfare. Fertility control programs aim to reduce the population size by reducing the population’s birth rate. Both no effect [36] and reductions in dog population size [37,38] have been reported following fertility control programs in India. In turn, a reduction in population size and increase in rabies vaccination following neutering and vaccination campaigns in free-roaming dog populations may reduce human bite and rabies cases. Reductions in both human bite cases [39] and human rabies cases [37,40,41] have been reported following neutering and vaccination campaigns in India. These impacts have been associated with varying intervention coverage (e.g., neutering coverage) and lengths of management.

Understanding the impact of CNVR is important for informing future dog population management efforts, so that we can optimise interventions and reduce the risks to humans and other animals that are associated with free-roaming dogs. Humane Society International (HSI; now operating as Humane World for Animals) is one of the largest animal protection and welfare organisations that promotes the humane treatment of animals worldwide and works to protect all animals through advocacy, education and direct care. Through effective dog population management programs, HSI aims to reduce the size of free-roaming dog populations, improve their welfare, reduce shelter intake and euthanasia, as well as to reduce the number of accidental pet owners through the rehoming of unwanted puppies. Between 2013 and 2018, HSI in partnership with the Jamshedpur Utility Services Company Limited (JUSCO), a TATA Steel company appointed authority, implemented a CNVR program in Jamshedpur, India. During this program, free-roaming dogs were mostly hand caught on the street by trained animal handlers, vaccinated against rabies, neutered, and returned to the capture location. Between 2013 and 2016, the program neutered and vaccinated a total of 20,915 dogs. Street surveys were also conducted by HSI between 2014 and 2018 to measure the impact of this intervention on the free-roaming dog population size, structure, health, and welfare.

This study investigated whether HSI’s CNVR program in Jamshedpur was associated with changes in the health and welfare of the local free-roaming dog population, and the overall size, structure, and reproductive potential of the population. The objectives of this study were to: (i) determine whether, when comparing the same site over time, CNVR programs were associated with a reduction in the probability of (a) dogs entering the CNVR clinic with infectious and non-infectious clinical conditions, (b) dogs entering the CNVR clinic pregnant, (c) observing puppies in the free-roaming dog population (i.e., changing the structure of the population to older individuals), and, (d) observing lactating females in the free-roaming dog population; and (ii) determine whether the count of dogs observed in street surveys significantly declined throughout the study period.

## 2. Methods

### 2.1. Ethics Statement

Ethical review was not required for this study, as we analysed secondary data obtained from street dog sterilisation programs conducted according to standard operating procedures. No animals were specifically handled for the purpose of this study; instead, we analysed this dataset retrospectively to investigate the potential impact of government-supported animal birth control initiatives. In accordance with the Animal Welfare Board of India and the Animal Birth Control Rules of 2001, the Humane Society International has established written agreements with the local municipalities to carry out their dog sterilisation programs.

### 2.2. Study sites

**Fig 1:**
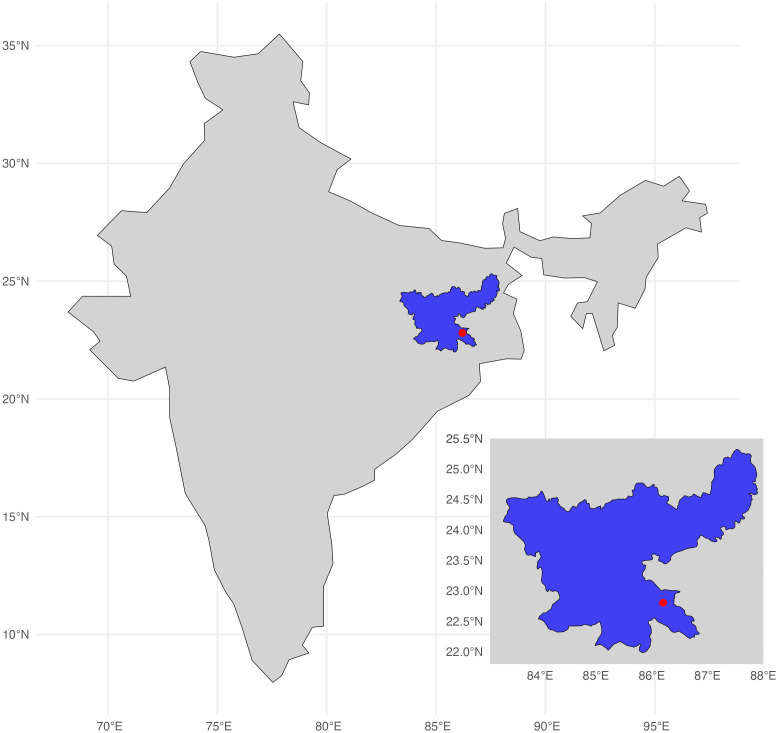
Inset map of India highlighting the state of Jharkhand in blue, with the location of the city of Jamshedpur indicated with a red dot. Map created using Natural Earth map data, and created in R using sf, ggplot2, and rnaturalearth packages [43].

This study investigated data collected by HSI as part of a CNVR programme conducted in Jamshedpur, India (Fig 1). Jamshedpur is the third biggest city in the state of Jharkhand, in north-eastern India. It has a tropical climate with mean daily temperatures between 12^o^C and 39^o^C and a wet season between June and October. The Greater Jamshedpur Metropolitan Region has a human population size of 1,339,438 [42]. The CNVR project focussed on the JUSCO run township area (Jamshedpur Central). The study area was split into the following sites: Adityapur, Baridih, Bistupur, Golmuri, Jugsalai, Mango I, Mango II, Sidhgora, Sonari, and Telco. All sites had direct CNVR intervention, apart from Adityapur, Mango I, and Mango II, which acted retrospectively as negative controls. Although sterilised dogs were observed in these control sites, their proportion was consistently lower compared to sites with direct intervention. Our analysis focussed on comparing sites with higher and lower percentages of sterilised dogs. The CNVR project was launched in 2013, in partnership with the Animal Health Foundation (an animal welfare organisation) and ran until 2017.

### 2.3. Data collection

Data on the free-roaming dog population was collected through street surveys conducted between May 2014 and December 2018. Clinical data was recorded from all dogs captured as part of the CNVR program between July 2013 and December 2016. Both clinical and street survey data were collected as part of the CNVR campaign by HSI staff members, and later shared with the authors for analyses.

#### 2.3.1. Street surveys.

Street surveys were carried out bi-annually in May and November between 2014 and 2016, and then annually in November/December in 2017 and 2018. All study sites were surveyed at least once on each sampling occasion (i.e., no study sites had missing surveys). Within each study site, survey routes of approximately 25–30 km in length were selected along existing pathways, this allowed us to compare dog demographic and health factors between sites of varying size. Distances between study sites varied between 1 and 10 km. Surveys were conducted by two surveyors simultaneously using motorbikes and were usually conducted in the early morning. Surveyors travelled along the pre-determined routes moving at no more than 15 km/hr. Every free-roaming dog was recorded, excluding those enclosed in private property or walking on a lead. The risk of double counting was reduced by covering fixed routes on motorbike at a consistent speed. These measures minimised the possibility of the same dog being recorded more than once during a survey. At each sampling occasion, the standard protocol for HSI was to conduct two surveys over two consecutive days. In 11% of the site/sampling occasions, only one survey was conducted due to constraints such as resources or unusual weather which would have affected the probability of observing dogs. A third survey was conducted in 8% of the occasions, when there was substantial variation between the first two counts or notable gaps in demographic data (e.g., unknown gender or sterilisation status).

Once a free-roaming dog was identified, information was collected by the surveyors using the mobile app OSM tracker to record each individual dog. Information on the dog’s age (adult: dogs that looked more than four months of age, or puppies: dogs that looked four months or younger), sex (male/female), lactation status (lactating/not lactating), and vaccination and neutering status (both determined by presence of ear notch) were determined visually and recorded [44]. It is important to note that the presence of an ear notch confirmed the dogs’ sterilisation status, but did not provide information on when the dog was last vaccinated against rabies. The presence of an ear notch instead indicates that a dog has been vaccinated at least once, when it was caught for sterilisation. In addition, the presence/absence of visible skin conditions was recorded, based on the presence/absence of hair loss, dermatitis, or visible ectoparasites. Body condition was recorded, based on a scale from one to five, relating to the dog’s body fat coverage (1 = emaciated, 2 = thin, 3 = normal, 4 = overweight, 5 = obese) [44,45].

#### 2.3.2. Clinical data.

Clinical data was collected by HSI veterinary clinic staff for all dogs captured as part of the CNVR program. All dogs were neutered and treated at a fixed clinic location. After dogs underwent surgery, they were kept in the clinic for a minimum of three days for post-operative care and monitoring. Clinical data included a description of the dog, how the dog was delivered to the clinic (from owner, caught by hand/net), date of operation, total length of stay in clinic, weight, reproductive status (in season/not in season; pregnant/not pregnant), treatment provided (vaccination; neutering; and Ivermectin administration), length of operation, and clinical conditions (canine distemper, mange, rabies, canine transmissible venereal tumour, and pregnancy). Dogs were vaccinated against rabies upon admission to the clinic as a one-time component of the CNVR programme. We investigated whether time since implementation of CNVR was associated with changes in the probability of dogs entering the clinic with canine distemper, mange, rabies, canine transmissible venereal tumours and, for the female dogs, pregnancies. Rabies and canine distemper were diagnosed symptomatically, and transmissible venereal tumour disease and mange were diagnosed by visible appearance by qualified veterinarians at the clinic.

### 2.4. Statistical analyses

All statistical analyses were run in a Bayesian analysis framework using the package **brms** version 2.14.4 [46], in R version 4.3.1 [47]. A negative binomial mixed effects model was fit to determine whether the number of dogs counted during street surveys decreased following CNVR intervention. Binomial mixed models were fit to assess whether the probabilities of observing: (i) sterilised dogs, (ii) puppies, or (iii) lactating females (for female subset of data) during street surveys decreased following CNVR intervention. To allow for overdispersion, we fit a beta-binomial model to assess whether the probability of observing dogs with skin conditions decreased following CNVR intervention. Models had demographic or health parameters as the outcome variables, and (i) survey number, (ii) CNVR (direct = study sites where dogs were caught, neutered, vaccinated, and released; non-direct = study sites without direct intervention, i.e., lower percentages of dogs that had been caught, neutered, vaccinated, and released); and (iii) month (beginning of year (May)/ end of year (November/December)) as predictor variables. Survey number was included so that May 2014 was survey one, November 2014 was survey two, May 2015 was survey three, and so on. Since only annual surveys were conducted in November/December in 2017 and 2018, the surveys normally scheduled for May (numbers seven and nine) were missing to account for the extended period between surveys. Survey site was included as a random intercept. As there may be different associations between the outcome variables for sites where CNVR was directly applied over time, we compared models with additive effects of predictor variables, and a model with an interactive effect between CNVR and survey time point. We determined the best fitting model using kfold cross-validation (10-folds) using the **kfold** function in the **brms** package [46]. Kfold cross-validation involves subsetting the data into approximately equal folds. The model was then trained and assessed k-times using different folds as the test set to determine model fit.

To investigate whether the CNVR intervention was associated with changes in the probabilities of dogs entering the CNVR clinic with infectious conditions (canine distemper, mange, rabies, and canine transmissible venereal tumour disease), Bernoulli logistic regression models were fit with each of the infectious conditions as the outcome variables and year, month (to account for non-linear seasonal dynamics, we fitted using a generalised additive model function in the brms package), age (adult/puppies, determined visually), and sex (male/female, determined visually) as predictor variables. To determine whether the CNVR intervention was associated with changes in the probabilities of females entering the CNVR clinic in a pregnant state, a Bernoulli logistic regression model was fitted on a subset of the data for females only, with pregnancy as the outcome variable and year, month (to account for non-linear seasonal dynamics, we fitted using a generalised additive model function in the **brms** package), and age (adult/puppies) as predictor variables. As there may be different associations between the outcome variable for adults/puppies across different sexes, for each of the clinical conditions, we compared models with both additive and interactive effects of age and sex (excluding pregnancy, as this analysis included a subset of the data for female dogs only). We also evaluated whether models were better fit including a seasonal trend by comparing models with and without month as a predictor variable. We determined the best fitting model using kfold cross-validation (10-folds).

All models were run with four chains, each with 2000 iterations (1000 used for warmup and 1000 for sampling). Thinning was set to one. The total number of post-warmup samples was 4000. Missing data was omitted from the statistical analysis (see supplementary information for details). All models had flat prior distributions. Model parameters were summarised by the mean and 95% credible intervals (CI; 95% most probable values). A significant effect was determined if the 95% credible intervals of the posterior distribution did not contain zero on the log odds or log scale. All predictor variables were centred, allowing for clearer interpretation of the results. Posterior distribution estimates were converted from logit scale to odds using exp(x), where *x* is the posterior value on the logit scale. All reported models converged (for all parameters Rhat ~ 1.00 and effective sample size >1000, see Supplementary information).

## 3. Results

### 3.1. Street surveys – descriptive statistical analysis

**Fig 2:**
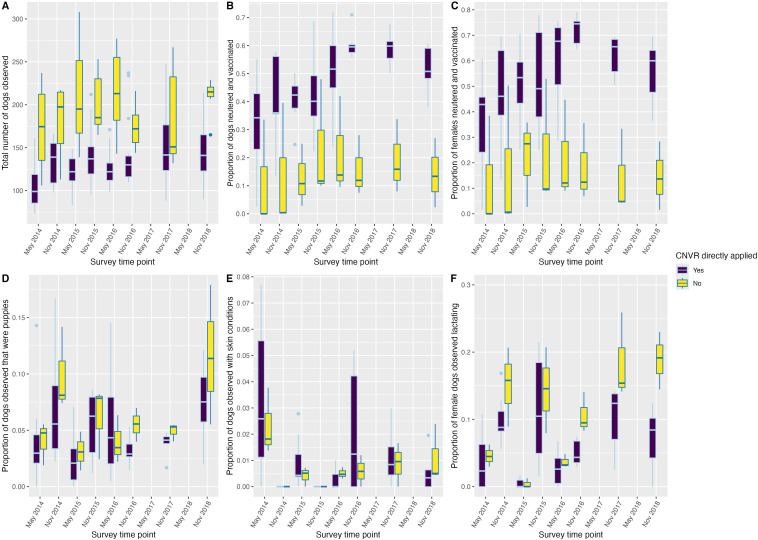
The total count of dogs observed during street surveys (A), and the proportion of dogs observed during street surveys that were neutered and vaccinated (B), female dogs neutered and vaccinated (C), puppies (D), observed with skin conditions (E), and females lactating (F) across survey period. **Boxplots illustrate the median and 25**^**th**^ **and 75**^**th**^ **percentiles.** Note: (i) that surveys were only conducted once per year in 2017 and 2018 in November/December and (ii) varying y-axis scales.

Table 1 outlines the demographic factors and health indicators of dogs observed in street surveys between May 2014 and December 2018. The mean ± SD number of dogs counted during surveys varied between 105 ± 30 and 214 ± 52 (Table 1). In study sites where CNVR was directly applied (i.e., the seven sites where dogs were caught, neutered, and released), the percentage of sterilised dogs observed increased from 27% to a maximum of 61% across the study period. This provides our best estimate of CNVR coverage in this study. In the three study sites with no direct CNVR the percentage of sterilised dogs observed varied between 13% and 26% across the study period, with no consistent trend. The percentage of both lactating females and puppies observed during surveys fluctuated annually with an increase in November and a decrease in May. The maximum percentage of puppies observed was 8% in study sites where CNVR was directly applied, and 11% in sites where CNVR was not directly applied. The maximum percentage of females that were observed to be lactating was 11% in study sites where CNVR was directly applied, and 19% in sites where CNVR was not directly applied. Most dogs had a normal body condition score. Few dogs were observed with skin conditions throughout the study period. Fig 2 shows the number of dogs counted, the proportion of dogs observed that were neutered, that were female and neutered, female and lactating, adults, and observed with a skin condition across all time points for sites where CNVR was directly (i.e., sites where dogs were caught, neutered, vaccinated and release), and not directly applied (i.e., sites where dogs were not caught, neutered, vaccinated, and released).

**Table 1:** Mean number (SD) of dogs counted during street surveys, and mean demographic and health factors of dogs observed between May 2014 and December 2018 for sites with direct CNVR (C; highlighted grey) and no direct CNVR (N) applied.

	C	N	C	N	C	N	C	N	C	N	C	N	C	N	C	N	C	N	C	N	C	N
Time point	Mean count (SD)	Mean count (SD)	Sterilised (%)		Females sterilised (%)		Puppies (%)		Skin Conditions (%)		Females lactating (%)		Body condition score (%)									
													1		2		3		4		5	
May-14	105 (30)	173 (59)	27	21	29	25	4	4	4	2	5	4	0.6	0.4	0	0	94.8	97.7	1.8	0.7	0.6	0.4
Nov-14	132 (26)	181 (43)	42	16	48	19	7	10	0	0	10	15	0	0	0	0	100	100	0	0	0	0
May-15	121 (20)	214 (86)	41	16	52	26	2	4	1	0	1	1	0	0.3	44.9	38.2	54.6	61.2	0.3	0.2	0	0.3
Nov-15	142 (32)	201 (37)	40	26	49	30	6	6	0	0	11	13	0	0	0	0	100	100	0	0	0	0
May-16	125 (21)	214 (52)	50	24	60	24	6	4	0	1	3	4	0.3	0.3	0.3	0.4	99.4	99	0	0	0.3	0.3
Nov-16	144 (43)	175 (27)	61	20	73	24	3	5	3	1	6	10	0	0	0.6	0.3	98.8	99.7	0.5	0	0	0
Nov-17	150 (42)	184 (61)	58	22	63	18	4	5	1	1	10	18	0	0	0	0.3	99.9	99.7	0	0	0	0
Dec-18	144 (35)	210 (21)	50	13	53	14	8	11	0	1	6	19	0	0.2	0.1	0.2	99.8	99.6	0	0	0	0.2

### 3.2. Street surveys – inferential statistical analysis

#### 3.2.1. Total count of dogs observed.

**Fig 3:**
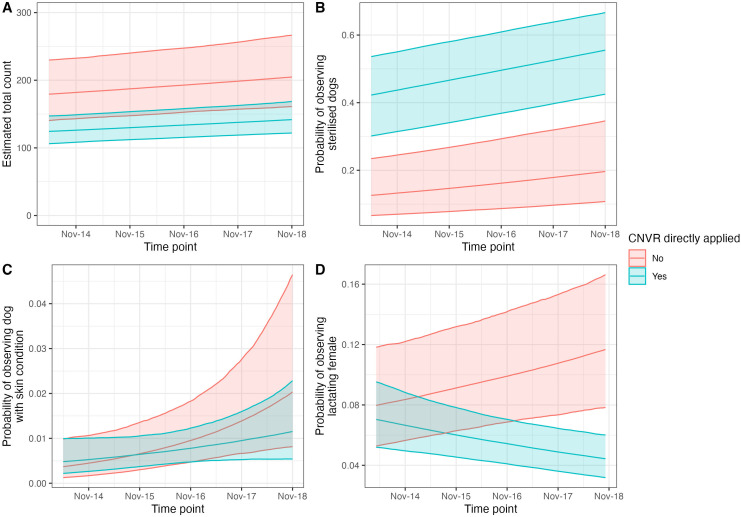
(A) Model estimated total count of dogs observed over time, (B) estimated probability of observing sterilised dogs over time, (C) observing a dog with a skin condition, and (D) observing lactating female for sites with CNVR directly applied and not. Ribbons show the 2.5 and 97.5 percentiles of the posterior distribution (95% CI).

The model with an additive effect between CNVR and survey timepoint was a better fit than an additive model (S1 File). Sites where CNVR had been directly applied had significantly lower counts of dogs (rate ratio 0.69, 95% CI 0.51–0.90). In sites where CNVR had been directly applied, the average model estimated count of dogs was 133 (95% CI 113–153), compared to 193 (95% CI 149–242) in areas where no direct CNVR was applied. The total count of dogs significantly increased over time (rate ratio 1.01, 95% CI 1.00–1.03; Fig 3A). There was no evidence of a significant effect of biannual survey month (May versus November/December) (rate ratio 1.01, 95% CI 0.998–1.021).

#### 3.2.2. Proportion of sterilised dogs.

The model with an additive effect between CNVR and survey timepoint was a better fit than an interactive effect (S1 File). The model estimated probability that an observed dog was sterilised significantly increased over time (OR 1.06, 95% CI 1.05–1.07; Fig 3), and there was a significantly higher probability of observing sterilised dogs in sites where CNVR had been directly applied (OR 5.64, 95% CI 1.59–11.00). The estimated probability of observing a sterilised dog in sites with direct CNVR was 0.46 (95% CI 0.35–0.57), compared to 0.15 (95% CI 0.05–0.16) in sites without direct CNVR. There was no evidence of significant associations between biannual survey month (May versus November/December) and the probability of observing sterilised dogs (OR 1.01, 95% CI 0.99–1.02).

#### 3.2.3. Proportion of puppies.

The model with an additive effect between CNVR and survey timepoint was a better fit than an interactive model (S1 File). The month of the survey (May or November/December) was significantly associated with the probability that an observed dog was a puppy (OR 1.07, 95% CI 1.04–1.10). In May, the model estimated probability that an observed dog was a puppy (0.04, 95% CI 0.03–0.5) was significantly lower than in November/December (0.06, 95% CI 0.05–0.07). There was no evidence of a significant association between the estimated probability that an observed dog was a puppy and survey time point (OR 1.02, 95% CI 0.999–1.042) or sites where CNVR had been applied directly (OR 0.77, 95% CI 0.51–1.04).

#### 3.2.4. Proportion of lactating females.

The model with an interactive effect between CNVR and survey timepoint was a better fit than an additive model (S1 File). The month of the survey (May or November) was significantly associated with the probability that an observed female dog was lactating (OR 1.29, 95% CI 1.23–1.35). In May, the model estimated probability that an observed female dog was lactating (0.03, 95% CI 0.02–0.03) was significantly lower than in November/December (0.11, 95% CI: 0.09–0.13). There was a significant interactive effect of CNVR and time point (OR 0.90, 95% CI 0.86–0.95; Fig 3D).

#### 3.2.5. Proportion of dogs with skin conditions.

The model with an additive effect between CNVR and survey timepoint was a better fit than an interactive effect (S1 File). The biannual survey month (May versus November/December) was significantly associated with the probability that an observed dog had a skin condition (OR 0.85, 95% CI 0.77–0.93). In May, the model estimated probability that an observed dog had a skin condition (0.02, 95% CI 0.009–0.025) was significantly higher than in November/December (0.007, 95% CI 0.004–0.011). The probability that an observed dog had a skin condition significantly increased over time (Fig 3C; OR 1.14, 95% CI 1.03–1.27). There was no evidence of significant associations between observing dogs with skin conditions and sites where CNVR had been directly applied (OR 0.87, 95% CI 0.27–3.51).

### 3.3. Clinical data – descriptive statistical analysis

**Fig 4:**
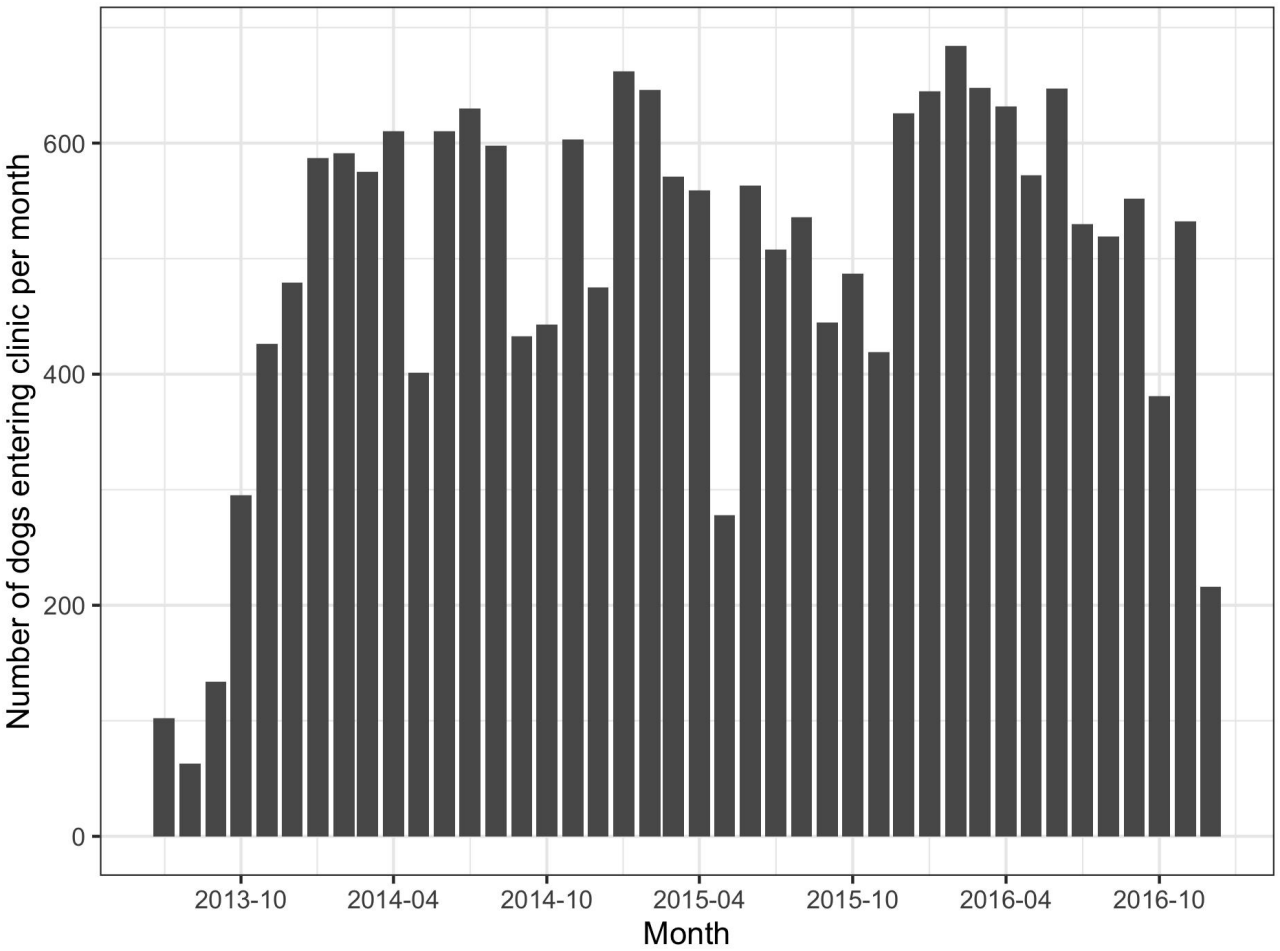
Number of dogs entering clinic per month as part of the Humane Society International’s Catch-Neuter-Vaccinate-Release intervention in Jamshedpur, India.

**Fig 5:**
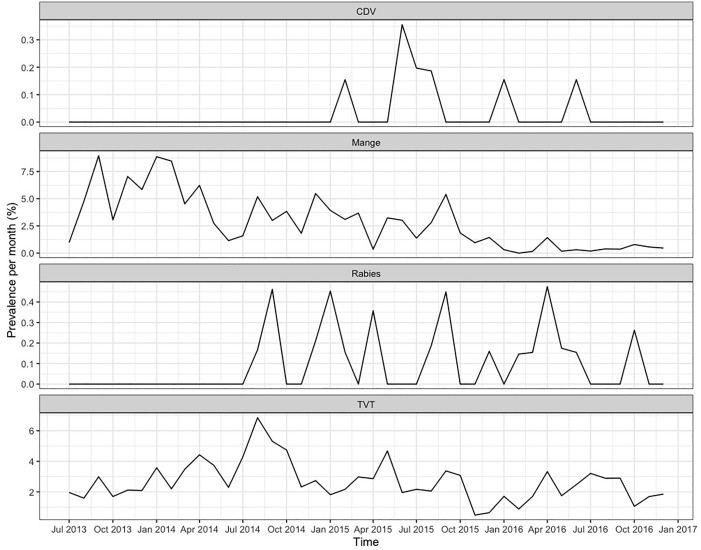
The percentages of dogs per month in Jamshedpur CNVR clinic that were diagnosed by visible appearance as positive for canine distemper (CDV), mange, rabies, and canine transmissible venereal tumour (TVT). Note the different y-axis scales per plot.

**Fig 6:**
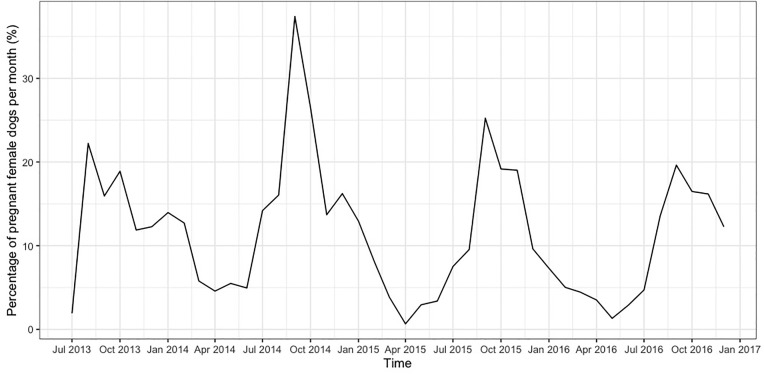
The percentages of female dogs per month entering the clinic in Jamshedpur CNVR clinic that were pregnant.

In total, 20,915 dogs were taken into the clinic to be neutered and vaccinated in the CNVR program in Jamshedpur between 4^th^ July 2013 and 13^th^ December 2016. A mean of 498 (± 155) dogs were neutered and vaccinated every month (Fig 4). Of these 13,062 (62%) were caught by hand, 7,448 (36%) were caught by net, 305 (1%) were brought in by owners, 85 (0.4%) were brought into the clinic by unreported method (missing information), and 15 (0.07%) were trapped. In total, the CNVR program neutered 10,459 (50%) females, 10,218 (49%) male dogs, and 238 (1%) dogs with missing information on their sex. The CNVR program neutered 13,087 (63%) adults, 7,590 (36%) puppies, and 238 (1%) dogs with missing information on their age. Fig 5 shows the percentages of dogs per month that were diagnosed by visible appearance as positive for canine distemper, mange, rabies, and canine transmissible venereal tumour. Fig 6 shows the percentages of female dogs per month that entered the clinic pregnant; as previously reported the percentages of pregnant dogs peaked each year in September/October [48].

### 3.4. Clinical data – inferential statistical analysis

#### 3.4.1. Proportion of dogs entering clinic with mange.

**Fig 7:**
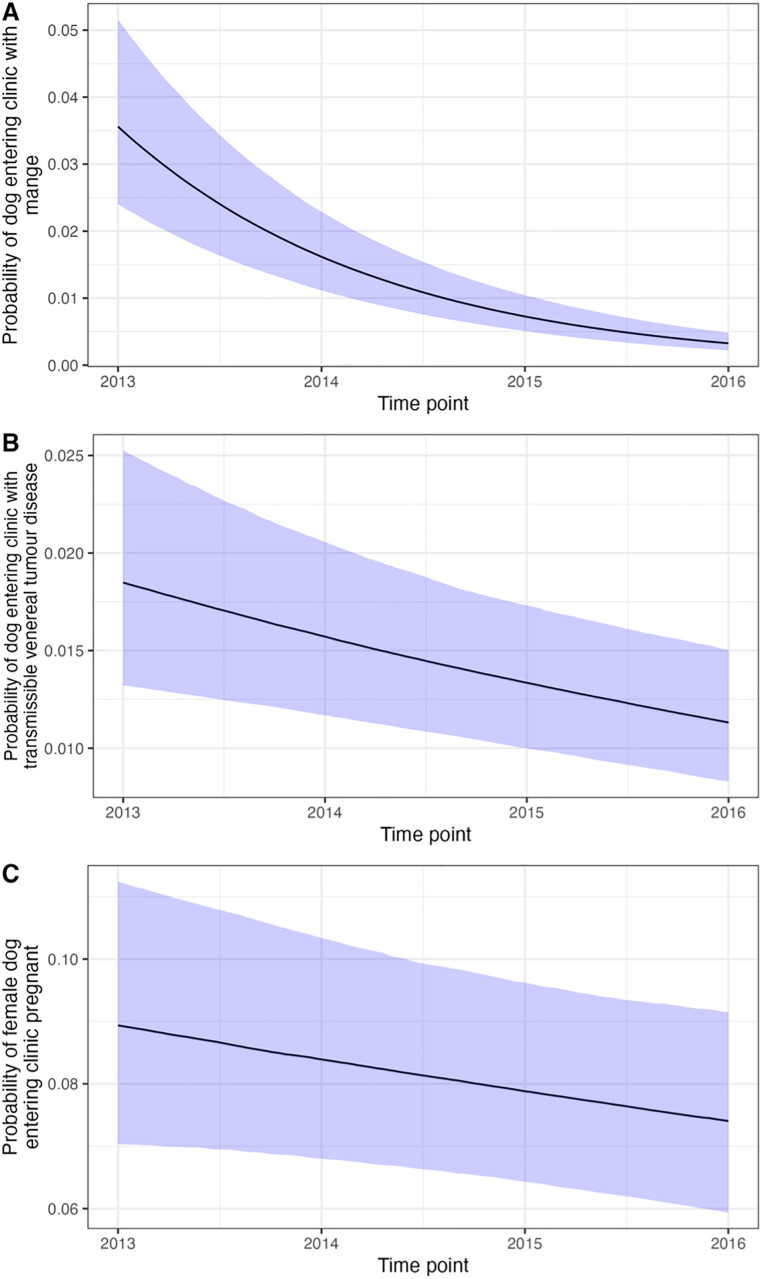
Statistical association between year and the probability of a dog entering the clinic with (A) mange, (B) transmissible venereal tumour disease, and (C) pregnant (female only). Estimated probabilities are averaged for all other predictor variables, apart from pregnancy, which reports the estimated effect for an adult dog. Ribbons show the 2.5 and 97.5 percentiles of the posterior distribution (95% CI).

**Fig 8:**
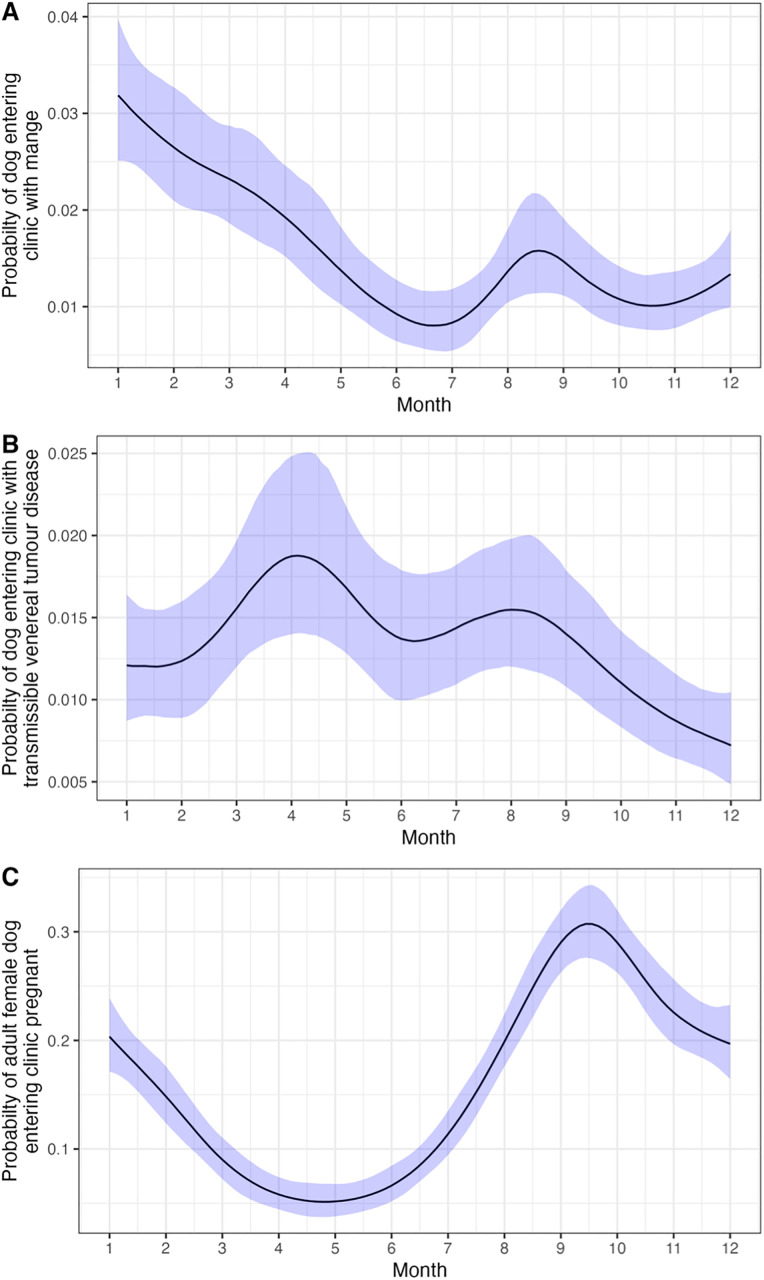
Statistical association between month and the probability of a dog entering the clinic with (A) mange, (B) transmissible venereal tumour disease, and (C) pregnant (female only). Estimated probabilities are centred for all other predictor variables, apart from the association between month and pregnancy, which reports the estimated effect for an adult dog. Ribbons show the 2.5 and 97.5 percentiles of the posterior distribution (95% CI).

The model with month of the year included and age and sex as additive fixed effects was best fitting (S1 File). The model estimated probability of dogs entering the clinic with mange significantly decreased with year since the start of the intervention (OR 0.45, 95% CI 0.40–0.50; Fig 7). There was a significant effect of month (mean and 95% credible intervals = 0.13, 95% CI 0.09–0.16; Fig 8). Female dogs (OR 0.63, 95% CI 0.52–0.74) had a significantly lower probability of entering the clinic with mange, female dogs had a 1.29% (95% CI 1–1.41% probability compared to 1.90% (95% CI 1.66–2.20%) for males. Adult dogs (OR 4.32, 95% CI 3.22–5.55) had a significantly higher probability of entering the clinic with mange, adult dogs had a 2.54% (95% CI 2.22–2.82%) probability compared to 0.61 (95% CI 0.46–0.78%) for puppies.

#### 3.4.2. Proportion of dogs entering clinic with canine transmissible venereal tumour.

The additive model with a seasonal component was best fitting (S1 File). The model estimated probability of dogs entering the clinic with canine transmissible venereal tumours significantly decreased with the year (OR 0.85, 95% CI 0.77–0.94; Fig 7). Adult dogs had a higher estimated probability of entering the clinic with canine transmissible venereal tumour (OR 16.24, 95% CI 9.30–24.05), adult dogs had a 3.44% (95% CI 3.11%−3.77%) probability of entering the clinic with canine transmissible venereal tumours, compared to 0.23% (95% CI 0.13%−0.33%) for puppies. Female dogs (OR 2.49, 95% CI 2.05–2.98) had a higher probability of entering the clinic with canine transmissible venereal tumours, female dogs had a 1.99% (95% CI 1.65%−2.36%) probability of entering the clinic with canine transmissible venereal tumours, compared to 0.82% (95% CI 0.63%−0.99%) for male dogs. The probability of dogs entering the clinic with canine transmissible venereal tumour disease was statistically significantly associated with the month of the year (mean and 95% credible intervals = 0.07, 95% CI 0.03–0.11; Fig 8).

#### 3.4.3. Proportion of dogs entering clinic with rabies.

The model with the interactive effect of sex and age, without a seasonal component was best fitting (S1 File). There were no significant statistical associations between the probability of dogs entering the clinic with rabies and year (OR 1.93, 95% CI 0.82–3.18), age (OR 1.03, 95% CI 0.16–2.39), sex (OR 1.12, 95% CI 0.18–2.55), or interactive effect of age and sex (OR 13.20, 95% CI 0.09–44.22).

#### 3.4.4. Proportion of dogs entering clinic with canine distemper.

The model with an additive effect of sex and age, without a seasonal component was best fitting (S1 File). There were no significant statistical associations between the probability of dogs entering the clinic with canine distemper and year (OR 1.89, 95% CI 0.43–4.19), age (OR 1.91, 95% CI 0.04–6.26), or sex (OR 20.58, 95% CI 0.13–57.79).

#### 3.4.5. Proportion of female dogs entering clinic pregnant.

The model with month of the year included was best fitting (S1 File). The probability of female dogs entering the clinic pregnant significantly decreased with time since the start of the intervention (Fig 7; OR 0.93, 95% CI 0.87–0.99). Adult females had a higher probability of entering the clinic pregnant (OR 33.25, 95% CI 20.49–47.23), adult females had a 13.42% (95% CI 12.56–14.35%) probability of entering the clinic pregnant, compared to 0.48% (95% CI 0.30–0.69%) for female puppies. There was a significant association between the month of the year and probability of entering the clinic pregnant (mean and 95% credible intervals = 0.137, 95% CI 0.109–0.167; Fig 8).

## 4. Discussion

This study determined whether the introduction of the CNVR program was associated with changes in (i) the health and welfare of the local free-roaming dog population, and (ii) the overall size, structure, and reproductive potential. We found that the probability of dogs entering the clinic with mange, canine transmissible venereal tumour disease, or pregnant significantly decreased with time since the start of the CNVR programme. In the street surveys of free-roaming dogs, the probability of observing sterilised dogs increased over time, and there was a higher probability of observing sterilised dogs in sites where CNVR had been directly applied. Despite the overall increase in sterilised dogs, survey counts significantly increased over time, although lower counts of dogs were observed in CNVR-treated sites. The age-structure observed in street surveys did not significantly change over time. In CNVR-treated sites, the probability of observing lactating females declined, while in non-treated sites it increased.

### 4.1. Health and welfare

Most dogs observed in the street surveys had normal body condition scores and few dogs had observable skin conditions. These results are similar to those reported by other studies in South Asia [49,50], although different to Totton *et al.* [30], who reported a high prevalence of dogs with low body conditions (70%) and skin conditions (69%) in Jodhpur, India. Free-roaming dogs are sustained from food primarily provided from people, either directly through deliberate feeding, or indirectly through human waste [22,51,52]. The normal body condition scores reported in this study suggest sufficient supply of food for this free-roaming dog population. Bhalla *et al.* [22] reported that bakeries, houses, and garbage piles are significant sources of food to free-roaming dogs in Bangalore, India.

We observed a small, but significant positive correlation between the probability of observing dogs with skin conditions and time since the beginning of the CNVR intervention, while no significant difference between CNVR versus non-CNVR treated sites were seen. Previous research has reported an increase in the prevalence of skin conditions following CNVR interventions [29,30]. This is possibly due to the conditions dogs were kept in the clinic both pre- and post-sterilisation, such as increased density of dogs that could facilitate the spread of infectious diseases, and whether antiparasitic treatment was administered. In this study, all dogs sterilised as part of the CNVR intervention were treated with Ivermectin, an antiparasitic drug.

In the clinical analysis, we observed a significant decrease in the probability of dogs entering the CNVR clinic with mange following time since the beginning of the CNVR intervention, suggesting a possible decrease in the prevalence of mange in the wider free-roaming dog population. By contrast, we observed an increasing trend in the prevalence of skin conditions in the street survey data. One explanation for the higher estimates from street surveys may be the differences in definitions: the clinical data only included mange, diagnosed visually by a veterinarian, whereas street surveys recorded any visible skin condition [44]. Observing skin conditions from distance in the street surveys has limitations. Dogs may have had skin conditions that were not visible, leading to false-negatives, or dogs may have been perceived to have visible skin conditions when they were healthy, leading to false-positives. These rates of false-positives and false-negatives are unknown. The administration of Ivermectin to all dogs sterilised in the CNVR program could influence the dynamics of parasitic diseases in the wider free-roaming dog population [29,30], although, given the differing trends in the street survey and clinical datasets, further research would be required to ascertain this relationship.

We observed a significant decrease in the probability of dogs entering the clinic with canine transmissible venereal tumour disease following time since the beginning of the CNVR intervention. Canine transmissible venereal tumour disease is usually transmitted during mating and is one of the few naturally transmissible cancers in mammals. It has an estimated global prevalence of around 1% [53], but may be as high as 15% in females in some free-roaming dog populations [54]. As sexually intact dogs mate more frequently, they are at higher risk of contracting this disease. If more dogs in the population are sterilised, the frequency of mating may reduce, therefore reducing the risk of exposure to this disease. We estimated that female dogs had a higher probability of canine transmissible venereal tumour infection (1.99%, 95% CI 1.65%, 2.36%), compared to males (0.82%, 95% CI 0.63%, 0.99%). This has been reported elsewhere and has been associated with the mating dynamics of free-roaming dogs [55,56]; males are sexually receptive all year and one infected male often mates with several females, leading to a higher prevalence in female dogs [57,58].

We did not observe a significant association between time since the beginning of the CNVR program and the probability of dogs entering the clinic with rabies or canine distemper. In general, the monthly prevalence of dogs observed in the clinic with either distemper or rabies was low, therefore there was low power to determine statistically significant associations. It is also worth noting that dogs were diagnosed symptomatically, limiting our confidence in the reported estimates. As dogs were vaccinated against rabies as part of the CNVR program, we may have expected to observe a decline in the number of dogs entering the clinic with rabies. It is estimated that 70% vaccination coverage of dog populations is required to prevent a rabies disease outbreak [59,60]; declines in canine and human rabies cases have been reported in areas following annual vaccination of 70% of the dog population [61,62]. Given the neutering and vaccination coverage was consistently below 60% in the study sites, a greater number of dogs may need to be vaccinated to observe a decline. Immunity towards rabies may also be lower, due to effects of waning vaccination if dogs are not regularly vaccinated. Mass vaccination delivered as part of CNVR efforts, where dogs receive only a single vaccine without follow-up, may have limited long-term impact on rabies control and offer low cost-effectiveness.

The sampling of dogs taken into the clinic for surgical sterilisation represents a convenience sample, and here we assume that this sample is representative of the wider population. In reality, only intact dogs (or those assumed to be intact) and those able to be captured are included in this convenience sample. This may bias our estimated prevalence of infectious diseases in the wider free-roaming dog population. For example, dogs with rabies may be less likely to be caught, leading to underestimation of the prevalence of rabies in the wider dog population. A cross-sectional sero-prevalence survey of the dog population for antibodies against infectious diseases of interest may provide less-biased estimates of associations between CNVR programs and the prevalence of infectious diseases. Surveys of this kind would require dogs to be caught and tested regardless of their neutering status (i.e., not only dogs taken into the clinic as part of the CNVR campaign). The processing of samples would be associated with increased cost, however the benefit would be a more accurate and representative understanding of the disease status within the wider dog population.

### 4.2. Population size, structure, and reproductive potential

The counts of dogs observed in the street significantly increased over time. This is possibly due to the low neutering coverages observed, which were often observed to be lower than 60%. CNVR aims to decrease the birth rate in the free-roaming dog population, with the hypothesis that it will result in stabilisation, eventually leading to a reduction in dog population size. Neutering coverages maintained at 65% and higher over several years have been associated with declines in free-roaming dog populations in Jaipur, Jodhpur and Ranchi in India [26,36,37]. Additionally, the human population size in Jamshedpur grew by approximately 2% annually, which may have increased resources for dogs or led to their migration, as dog population size often correlates to human population size.

If CNVR impacts dog population size, we might expect age structure changes prior to observing population decline. We observed no significant change in adult and puppy proportions over time, including in CNVR-treated sites. This might be due to the low sterilisation coverage. We observed a small difference in age structure depending on the month of the survey: 6% (95% CI 5–7%) of the dogs observed in street surveys in November were puppies, compared to 4% (95% CI 3–5%) in May. Although dogs generally do not exhibit seasonal breeding, in India, dogs have one clear breeding season [48,63]. This season begins after the rainy season and high proportions of puppies are often observed at the end/beginning of the year. The age structures observed in this study align with those in Jodhpur and Ranchi [26,36], though we observed fewer puppies than in Maharashtra, India [18,64], where puppies comprised 15–30% of the population. These variations may reflect differences in CNVR interventions, or in visual age estimation methods. Juvenile dogs, particularly young puppies, may have a lower detection probability. For example, puppies may be hidden and less likely to be observed during street surveys, meaning proportions of adults and puppies observed during street surveys might not reflect the true age structure in the population, possibly making it challenging to observe changes in the age structure over time.

Although we did not observe a decline in the numbers of dogs counted, or a change in the age structure, we observed a significant decrease in the probability of female dogs entering the clinic pregnant with time since the start of the intervention. If CNVR has reduced the birth rate, we may expect to see fewer pregnant females in the clinic if there are fewer intact dogs in the population. We also observed an interactive effect between time since the beginning of the intervention, whether CNVR was directly applied and the probability of observing lactating females. The probability of observing lactating females increased in areas where CNVR was not directly applied, compared to the probability decreasing in areas with direct CNVR intervention. Perhaps over a longer time-frame and with neutering coverage maintained above a critical threshold, we would observe a reduction in the free-roaming dog population size also.

### 4.3. Study limitations

In this study, we compared population counts observed during street surveys through time. Simple count methods do not provide an estimate of the total population size, as this method does not account for imperfect detection of individuals. Instead, simple counts may act as an indicator of population size, showing relative trends over time. Simple count methods are logistically easier but their usefulness in tracking trends depends on whether they are directly proportional to the true population size [65,66]. This means that the probability of detecting individuals must be constant across survey time points. It is unclear if this assumption is true for free-roaming dog populations. Variation in detection probability has been associated with the time of day, day of the week, and time of year [50,67,68]. This may be controlled by ensuring surveys are conducted across consistent time, days, and months. Variation in detection probability due to other factors has been reported, such as days with food markets or festivals [68,69], and environmental conditions, such as wind velocity and ambient temperature [64]. Estimates of population size using mark-resight techniques would avoid these issues, and allow organisations involved in dog population management to plan their interventions to ensure high neutering and vaccination coverage.

We also acknowledge that more frequent street surveys conducted over a longer duration of years would likely have strengthened the robustness of this study’s findings. However, the frequency and duration of data collected were influenced by the availability of resources and logistical feasibility.

In the street surveys, dogs were characterised as adults or puppies through observation, using the criteria of whether a dog looked to be less than four months of age as a threshold. Defining age visually in this way does not provide accurate estimates. Dogs may have been categorised as puppies that were older than four months, and dogs older than four months may have been recorded as puppies. However, defining age in this way allowed us to distinguish visibly older and younger dogs for our analysis. While more accurate methods of age determination could provide more accurate estimates of changes in age structure over time, the use of a visual cut-off at approximately four months provided a practical and resource-efficient means of categorising dogs in a field setting.

Additionally, although we compared sites with and without directly applied CNVR, neutered dogs were observed in both locations, with increasing proportions observed during 2015–2016. This suggests movement of dogs between sites, and although lower numbers of neutered dogs were observed in sites without CNVR, this will have reduced our ability to detect an effect of CNVR. Study designs, such as stepped-wedge interventions, where interventions are applied systematically to areas over time, with each area acting as a control at the beginning may be beneficial for future investigating the impact of dog population management interventions. This study design also overcomes the ethical challenge of keeping some areas as controls.

## 5. Conclusions and future recommendations

Over recent years, there has been increasing appreciation of the importance of measuring the impact of existing dog population management programmes [27,70]. This is important for gaining insight into the factors that influence the success of management strategies. The information presented in this study contributes to this growing body of research. Our results suggest that a low coverage of around 60% may be associated with a reduction in mange and canine transmissible venereal tumour disease, and a reduction in the proportion of lactating females, but is not enough to reduce the population size. This knowledge can help guide future dog population management efforts, suggesting organisations should aim to maintain high sterilisation coverages. Further monitoring and evaluation of CNVR program impacts are required to determine optimal coverages required to reduce population size.

We observed clear seasonality in our study sites in Jamshedpur. This suggests that to optimise CNVR interventions, maximising sterilisation coverage prior to the breeding season could potentially allow efficient and effective high intervention coverages to be maintained during the critical breeding period. This approach may reduce the resources and costs required, such as staffing and facilities. The impact of timing CNVR interventions with the seasonal breeding cycle of Indian free-roaming dogs requires further investigation. For example, modelling the effect of timing interventions prior to the breeding season on intervention costs and population size.

## Supporting information

S1 File Supporting information for missing data, model comparisons and model outputs. (DOCX)
