# Supplementary material for: Changes in free-roaming dog population demographics and health associated with a catch-neuter-vaccinate-release program in Jamshedpur, India
Source: PLoS One. 2025 Oct 7;20(10):e0317636. doi: 10.1371/journal.pone.0317636 (PMC12503238; doi:10.1371/journal.pone.0317636)
Supplement: S1 File — (DOCX) [file pone.0317636.s001.docx]

**Supporting information**

1. Missing data

There was no missing data for the street surveys. In the clinical data, there was missing data for date of surgery (predictor variable time point) for two dogs (0.01%), and for age and sex for 238 dogs (1.14%).

1. Model comparison

Table S1. Model comparison results. Best fitting models are highlighted in grey.

|  |  |  | Elpd kfold estimate | Standard error | Elpd_diff | se_diff |
| --- | --- | --- | --- | --- | --- | --- |
| Street survey analysis | Total count model | Additive | -748.9 | 11.8 | 0 | 0 |
|  |  | Interactive | -755.0 | 12.1 | -6.1 | 2.3 |
|  | Sterilised model | Additive | -1508.9 | 128.6 | 0.0 | 0.0 |
|  |  | Interactive | -1516.1 | 147.1 | -36.5 | 186.7 |
|  | Puppy model | Additive | -613.9 | 39.5 | 0.0 | 0.0 |
|  |  | Interactive | -620.1 | 42.0 | -6.1 | 8.1 |
|  | Lactating females model | Additive | -394.1 | 23.6 | -6.7 | 2.9 |
|  |  | Interactive | -387.4 | 20.7 | 0.0 | 0.0 |
|  | Skin conditions model | Additive | -232.9 | 17.8 | 0.0 | 0.0 |
|  |  | Interactive | -234.6 | 18.1 | -1.7 | -1.6 |
| Clinical analysis | Mange model | Additive with season | -2255.7 | 75.5 | 0.0 | 0.0 |
|  |  | Additive without season | -2295.9 | 76.4 | -40.2 | 9.6 |
|  |  | Interactive with season | Did not converge | | | |
|  |  | Interactive without season | -2294.4 | 76.3 | -38.7 | 9.7 |
|  | TVT model | Additive with season | -2238.9 | 74.9 | 0.0 | 0.0 |
|  |  | Additive without season | -2249.0 | 75.1 | -10.1 | 5.4 |
|  |  | Interactive with season | -2241.2 | 75.0 | -2.3 | 2.1 |
|  |  | Interactive without season | -2253.1 | 75.4 | -14.2 | 5.8 |
|  | Rabies model | Additive with season | Did not converge | | | |
|  |  | Additive without season | -127.0 | 28.0 | 2.0 | 2.2 |
|  |  | Interactive with season | Did not converge | | | |
|  |  | Interactive without season | -125.0 | 28.4 | 0 | 0 |
|  | CDV model | Additive with season | Did not converge | | | |
|  |  | Additive without season | NA | | | |
|  |  | Interactive with season | Did not converge | | | |
|  |  | Interactive without season | Did not converge | | | |
|  | Pregnancy | Including season | -2911.8 | 56.6 | 0.0 | 0.0 |
|  |  | Excluding season | -3077.2 | 57.7 | -165.4 | 17.4 |

* We determine models did not converge given the Rhat >1 and ESS < 1000. We exclude these from the model comparison.

1. Model outputs

Note that all model outputs report centred predictor variables.

Table S2. Population count model outputs (street survey data). Significant effects are highlighted in bold.

|  | **Estimate** | **Est.Error** | **l-95% CI** | **u-95% CI** | **Rhat** | **Bulk_ESS** | **Tail_ESS** |
| --- | --- | --- | --- | --- | --- | --- | --- |
| **Intercept** | 4.996 | 0.068 | 4.865 | 5.13 | 1.005 | 1300.485 | 1474.737 |
| **Direct CNVR** | **-0.367** | **0.142** | **-0.657** | **-0.088** | **1.001** | **1267.241** | **1706.105** |
| **Time point** | **0.015** | **0.006** | **0.003** | **0.026** | **1.000** | **3680.305** | **2837.771** |
| **Month** | 0.01 | 0.006 | -0.002 | 0.021 | 1.001 | 3584.834 | 2366.971 |

Table S3. Sterilised model outputs (street survey data). Significant effects are highlighted in bold.

|  | **Estimate** | **Est.Error** | **l-95% CI** | **u-95% CI** | **Rhat** | **Bulk_ESS** | **Tail_ESS** |
| --- | --- | --- | --- | --- | --- | --- | --- |
| **Intercept** | -0.639 | 0.217 | -1.063 | -0.217 | 1.006 | 919.353 | 1063.975 |
| **Direct CNVR** | **1.624** | **0.461** | **0.737** | **2.531** | **1.007** | **1082.57** | **1229.066** |
| **Time point** | **0.059** | **0.006** | **0.049** | **0.071** | **1.001** | **2544.84** | **2225.452** |
| **Month** | 0.008 | 0.006 | -0.004 | 0.02 | 1.001 | 2688.933 | 2308.854 |

Table S4. Puppy (vs adult) model outputs (street survey data). Significant effects are highlighted in bold.

|  | **Estimate** | **Est.Error** | **l-95% CI** | **u-95% CI** | **Rhat** | **Bulk_ESS** | **Tail_ESS** |
| --- | --- | --- | --- | --- | --- | --- | --- |
| **Intercept** | -2.857 | 0.085 | -3.027 | -2.683 | 1.003 | 1332.766 | 1835.838 |
| **Direct CNVR** | -0.282 | 0.178 | -0.642 | 0.074 | 1.002 | 1176.506 | 1568.841 |
| **Time point** | 0.021 | 0.011 | 0 | 0.042 | 1.001 | 3204.021 | 2903.786 |
| **Month** | **0.07** | **0.013** | **0.046** | **0.094** | **1.001** | **3167.35** | **2904.23** |

Table S5. Skin condition model outputs (street survey data). Significant effects are highlighted in bold.

|  | **Estimate** | **Est.Error** | **l-95% CI** | **u-95% CI** | **Rhat** | **Bulk_ESS** | **Tail_ESS** |
| --- | --- | --- | --- | --- | --- | --- | --- |
| **Intercept** | -4.845 | 0.212 | -5.278 | -4.433 | 1.000 | 2335.486 | 2161.686 |
| **Direct CNVR** | -0.208 | 0.377 | -0.964 | 0.549 | 1.001 | 2076.992 | 2186.484 |
| **Month** | **-0.168** | **0.048** | **-0.265** | **-0.075** | **1.001** | **3164.65** | **3137.06** |
| **Time point** | **0.132** | **0.052** | **0.03** | **0.236** | **1.000** | **3529.038** | **3038.766** |

Table S6. Lactating model outputs (street survey data). Statistically significant associations are highlighted in bold.

|  | **Estimate** | **Est.Error** | **l-95% CI** | **u-95% CI** | **Rhat** | **Bulk_ESS** | **Tail_ESS** |
| --- | --- | --- | --- | --- | --- | --- | --- |
| **Intercept** | -2.578 | 0.116 | -2.817 | -2.347 | 1.003 | 1392.381 | 1805.365 |
| **Direct CNVR** | **-0.586** | **0.245** | **-1.099** | **-0.104** | **1.006** | **1261.754** | **1879.641** |
| **Time point** | -0.017 | 0.014 | -0.045 | 0.012 | 1.002 | 3001.522 | 2564.79 |
| **Month** | **0.257** | **0.022** | **0.215** | **0.303** | **1.001** | **3116.984** | **2854.253** |
| **Direct CNVR : time point_** | **-0.102** | **0.027** | **-0.154** | **-0.047** | **1.000** | **3374.014** | **2616.373** |

Table S7. Mange model outputs (clinical data). Significant effects are highlighted in bold.

|  | **Estimate** | **Est.Error** | **l-95% CI** | **u-95% CI** | **Rhat** | **Bulk_ESS** | **Tail_ESS** |
| --- | --- | --- | --- | --- | --- | --- | --- |
| **Intercept** | 1620.08 | 112.813 | 1391.183 | 1836.096 | 1.001 | 3101.556 | 2462.085 |
| **Year** | **-0.806** | **0.056** | **-0.914** | **-0.693** | **1.001** | **3100.713** | **2462.085** |
| **Adult** | **1.452** | **0.137** | **1.195** | **1.729** | **1.000** | **3570.072** | **2900.033** |
| **Female** | **-0.469** | **0.09** | **-0.644** | **-0.295** | **1.001** | **4185.674** | **2868.06** |
| **sseasonal_trend_1** | **0.128** | **0.018** | **0.092** | **0.164** | **1.000** | **3550.154** | **2589.707** |

Table S8. Canine transmissible venereal tumour disease model outputs (clinical data). Significant effects are highlighted in bold.

|  | **Estimate** | **Est.Error** | **l-95% CI** | **u-95% CI** | **Rhat** | **Bulk_ESS** | **Tail_ESS** |
| --- | --- | --- | --- | --- | --- | --- | --- |
| **Intercept** | -4.349 | 0.091 | -4.537 | -4.178 | 1.002 | 2424.684 | 2469.627 |
| **Year** | **-0.165** | **0.051** | **-0.267** | **-0.065** | **1.001** | **3745.631** | **2780.045** |
| **Adult** | **2.76** | **0.232** | **2.325** | **3.244** | **1.001** | **2428.434** | **2406.776** |
| **Female** | **0.906** | **0.093** | **0.726** | **1.099** | **1.002** | **3839.591** | **2791.17** |
| **seasonal_trend** | **0.067** | **0.021** | **0.026** | **0.11** | **1** | **4000.957** | **2918.305** |

Table S9. Rabies model outputs (clinical data). Significant effects are highlighted in bold.

|  | **Estimate** | **Est.Error** | **l-95% CI** | **u-95% CI** | **Rhat** | **Bulk_ESS** | **Tail_ESS** |
| --- | --- | --- | --- | --- | --- | --- | --- |
| **Intercept** | -7.62 | 0.332 | -8.322 | -7.024 | 1.002 | 2914.51 | 2753.491 |
| **Year** | 0.605 | 0.324 | -0.005 | 1.258 | 1.001 | 3439.362 | 2790.77 |
| **Adult** | -0.151 | 0.604 | -1.326 | 1.061 | 1.001 | 3200.227 | 2716.427 |
| **Female** | -0.07 | 0.598 | -1.268 | 1.127 | 1 | 3242.651 | 2751.836 |
| **Adult:Female** | 1.79 | 1.183 | -0.371 | 4.221 | 1.002 | 2987.911 | 2595.603 |

Table S10. Canine distemper model outputs (clinical data). Significant effects are highlighted in bold.

|  | **Estimate** | **Est.Error** | **l-95% CI** | **u-95% CI** | **Rhat** | **Bulk_ESS** | **Tail_ESS** |
| --- | --- | --- | --- | --- | --- | --- | --- |
| **Intercept** | -9.071 | 0.722 | -10.763 | -7.928 | 1.001 | 1348.051 | 1403.708 |
| **Year** | 0.464 | 0.556 | -0.545 | 1.657 | 1.001 | 2478.569 | 1918.418 |
| **Adult** | 0.105 | 0.997 | -1.816 | 2.194 | 1.001 | 3023.673 | 2636.948 |
| **Female** | 1.669 | 1.307 | -0.453 | 4.824 | 1.001 | 1487.434 | 1394.494 |

Table S11. Pregnancy model outputs (clinical data). Significant effects are highlighted in bold.

|  | **Estimate** | **Est.Error** | **l-95% CI** | **u-95% CI** | **Rhat** | **Bulk_ESS** | **Tail_ESS** |
| --- | --- | --- | --- | --- | --- | --- | --- |
| **Intercept** | -3.143 | 0.084 | -3.315 | -2.982 | 1.001 | 2229.331 | 2344.648 |
| **Year** | **-0.068** | **0.035** | **-0.14** | **0.000** | **1.001** | **4207.524** | **2634.431** |
| **Adult** | **3.482** | **0.207** | **3.088** | **3.912** | **1.002** | **2001.798** | **2278.516** |
| **seasonal_trend** | **0.137** | **0.015** | **0.108** | **0.167** | **1.001** | **3974.215** | **3124.81** |
